# A prospective study evaluating an artificial intelligence-based system for withdrawal time measurement

**DOI:** 10.1055/a-2721-6798

**Published:** 2025-11-20

**Authors:** Ioannis Kafetzis, Philipp Sodmann, Bianca-Elena Herghelegiu, Michela Pauletti, Markus Brand, Katrin Schöttker, Wolfram G. Zoller, Jörg Albert, Alexander Meining, Alexander Hann

**Affiliations:** 1 Interventional and Experimental Endoscopy (InExEn), Department of Internal Medicine II, University Hospital Würzburg, Würzburg, Germany; 2 Department of Internal Medicine and Gastroenterology, Katharinenhospital, Stuttgart, Germany

## Abstract

**Background:**

Withdrawal time has emerged as a critical quality measure in colonoscopy for colorectal cancer screening. Owing to the high variability in calculating withdrawal time, recent research has explored the use of artificial intelligence (AI) to standardize this process, but prospective validation is lacking.

**Methods:**

This prospective, superiority trial compared the accuracy of AI-assisted withdrawal time calculation with that of physicians during routine colonoscopy from December 2023 to March 2024. The gold standard was obtained via manual, frame-by-frame annotation of the examination video recordings. The AI also automatically generated an image report, which was qualitatively assessed by four endoscopists.

**Results:**

126 patients were analyzed. The proposed AI system demonstrated a significantly lower mean absolute error (MAE) in estimating withdrawal time compared with physicians (2.2 vs. 4.2 minutes; P < 0.001). This was attributed to examinations containing endoscopic interventions, where the AI had significantly lower MAE compared with physicians (2.1 vs. 5.2; P < 0.001). The MAE was comparable in the absence of interventions (2.3 vs. 2.3; P = 0.52). High-quality image reports were generated by the AI system; 97% were assessed as showing satisfactory timeline representation and 81% achieved overall satisfaction.

**Conclusion:**

Our study demonstrated the superiority of an AI system in calculating withdrawal time during colonoscopy compared with physicians, providing significant improvements, especially in examinations involving interventions. This work demonstrates the promise of AI in streamlining clinical workflows.

## Abstract (de)

**Graphical Abstract:**

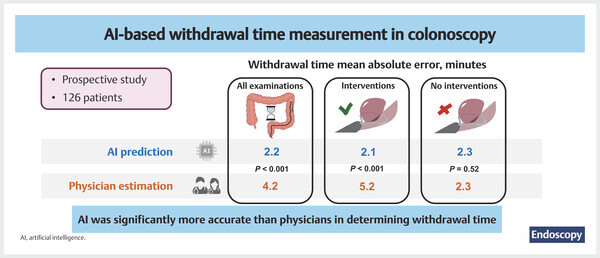

## Introduction

Colonoscopy has established itself as a cornerstone in reducing incidence and mortality rates of colorectal cancer 1 2 . To enhance consistency and quality in colonoscopy performance, various quality indicators 3 and guidelines regarding its proper execution and reporting have been introduced. Withdrawal time has emerged as a critical metric due to its inverse correlation with adenoma detection rate 4 5 and in turn, with the risk of colorectal cancer and death 6 .

The standard minimum duration for the withdrawal phase in colonoscopy is 6 minutes 7 8 9 . However, recent research has explored the implications of shorter 10 or longer 11 12 withdrawal duration on adenoma detection rate and other quality metrics. This is further supported by the observation that up to 13 minutes, a 1-minute increase in withdrawal time can increase adenoma detection rate by 6% 11 and the recent extension of the recommended withdrawal time from 6 to 8 minutes in the American quality indicators for colonoscopy 13 . Additionally, actively monitoring withdrawal time during colonoscopy has been shown to be beneficial to examination quality 14 . These findings have sparked interest in developing automated methods for calculating withdrawal time, improving accuracy, and reducing variability.

As artificial intelligence (AI) continues to integrate into clinical workflows in gastroenterology, various AI-based solutions for both commercial and research applications have been developed 15 16 . The majority of these tools focus on screening colonoscopy and particularly in polyp detection 17 18 19 , characterization 20 21 , and sizing 22 23 24 25 . Additionally, there is a growing interest in the development of AI-powered tools for documentation and withdrawal time calculation 26 27 28 29 . Furthermore, the use of speedometers has been investigated to monitor the speed and duration of withdrawal 30 . Recently, AI was utilized in defining novel quality metrics for withdrawal time, namely the effective withdrawal time 31 . Despite promising results from retrospective evaluations, prospective validation in real-world clinical settings is still necessary to establish compatibility with clinical routine.

In previous work, we investigated AI-based identification of withdrawal time in a retrospective study 28 . A multiclassification AI tool was used to identify anatomical landmarks, endoscopic findings, and resections in video recordings from colonoscopies. The predictions were then combined to determine the start and end of the withdrawal phase, as well as the duration of polypectomies. Additionally, the proposed method was able to generate an image report for the examination automatically.

This study presents the results of a prospective, superiority clinical trial comparing the performance of AI and physicians in estimating withdrawal time during colonoscopy. In addition to withdrawal time calculation, the AI photodocumented anatomical landmarks, polyps, and polypectomies, generating an image report for each examination. The main outcome of the study was the mean absolute error for the AI and physicians in withdrawal time calculation. Secondary outcomes included a subgroup analysis examining withdrawal time estimation errors in examinations with and without polypectomies, and an assessment of expert satisfaction with automatically generated reports. There are several differences between the current and our previous work. First, this was a prospective evaluation. Additionally, a new AI model was trained, following the novel architecture proposed in Kafetzis et al. 32 . Furthermore, the method was extended to more effectively estimate the beginning of withdrawal and the duration of polypectomies. Finally, the selection of images to be added to the automatically generated report was updated to give preference to higher-quality images.

## Methods

### Study design

This prospective, superiority trial compared the absolute error in calculating withdrawal time between AI and physicians. To reduce bias, consecutive individuals scheduled for colonoscopy were enrolled from December 2023 to March 2024 in a university hospital in Germany. Individuals with prior segmental colon resection, known inflammatory bowel disease, familial adenomatous polyposis syndrome, or previous radiation were excluded from the trial. According to standard procedure, patients were prepared using split-dose bowel preparation and were advised to follow a low-fiber diet, followed by a clear liquid diet the day before the colonoscopy.

Physicians estimated the withdrawal time empirically as usual and reported it at the end of the examination. To maintain the integrity of clinical routine, the study did not impose any specific guidelines or requirements regarding how physicians assessed the withdrawal time. Each endoscopy room was equipped with a standard wall-mounted clock, clearly visible to the examiner throughout the procedure. Physicians could also instruct the assisting nurse to mark the start of withdrawal using a designated button in the clinical documentation software, which also enabled recording of the end of the withdrawal phase. The physician’s assessment of the withdrawal time was obtained from the examination report in the clinic information system.

AI performed real-time analysis of the image content in the background throughout the examination, generating frame-by-frame predictions to identify anatomical landmarks, lesions, and interventions. Directly after the examination, these predictions were processed to determine the withdrawal time assessment and to generate the image report. To ensure unbiased results, examiners were blinded to any model outputs.

Patients were assessed independently by both the AI and physicians, making randomization unnecessary. The sample size was determined based on the results of our previous study 28 , to achieve 90% power and a significance level of 5%. Taking patient dropouts and potential technical issues into consideration, a total of 138 patients were to be recruited.

The gold standard reference withdrawal time for each examination was obtained by manual, frame-by-frame annotation. The beginning of withdrawal was annotated as the last visible frame of either the appendiceal orifice or the ileocecal valve. The end of withdrawal was marked as the last frame inside the patient’s body. Furthermore, the beginning and end frames of polypectomies and biopsies were marked. Frame numbers were converted to time based on the frame rate of the recording.

### AI-estimated withdrawal time

The AI assessed each frame of the colonoscopy video in real time to evaluate image quality, identify anatomical landmarks such as the cecum, appendiceal orifice, and ileocecal valve, and detect lesions and endoscopic instruments. The system also collected indicative images from these identified features, storing outputs for each frame to determine the start and end of withdrawal and total duration of any interventions. More explicitly, the start of withdrawal was set as the last frame where the cecum, ileocecal valve, or ileum was identified. In cases where none of these landmarks were identified, smoothing of the AI predictions was used to estimate the beginning of withdrawal. The end of withdrawal was set as the last frame inside the patient’s body. Finally, interventions were identified when a lesion or an endoscopic instrument was identified. The method is presented in detail in the **Supplementary Methods** in the online-only Supplementary Material. These identifications were required to be above a minimum duration, to reduce noise.

Combining the above stages with the frame rate of the video, the total withdrawal duration was estimated as the time from beginning to end of withdrawal. Finally, the corrected withdrawal time was obtained by subtracting the duration of endoscopic interventions from the total withdrawal duration. These data enabled the generation of a detailed timeline for the examination, breaking it down into three phases: insertion, inspection of the cecum, and withdrawal.

**Fig. 1:**
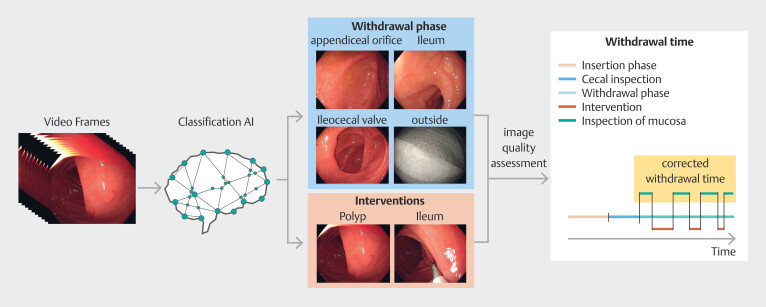
Schematic of the artificial intelligence (AI) method for withdrawal time estimation. The frames are passed through the AI system, which predicts the presence of landmarks as well as endoscopic procedures. Through these predictions, the total duration of the withdrawal phase and procedures is determined. Their difference is the actual withdrawal time. Additionally, the AI assesses images for their quality. Using predictions, high-quality, informative frames are collected and used to automatically generate an image report for the examination.

In addition to the timeline, collected images enabled the generation of an examination report, containing identified landmarks, polyp inspection, and interventions. The proposed method is shown in Fig. 1 . The report spans multiple pages, which follow a consistent format. The top section features a timeline, visualizing examination phases, and time intervals of polyp identifications and resections. The lower section is divided into three columns. On the first page, the left-most column is dedicated to images of anatomical landmarks. The remaining columns document identified polyps. For each polyp, the system aims to provide a clear image of the lesion, inspection with advanced imaging, and an image showing the polyp alongside the resection instrument, the latter two when available.

### Study outcomes and statistics

The primary outcome was the mean absolute error (MAE) in withdrawal time estimated by AI and physicians. Secondary outcomes included a subgroup analysis for examinations with and without interventions. Additional subgroup analysis based on the type of interventions was also undertaken, grouping examinations into those without interventions, with biopsies but no polypectomies, with polypectomies and no biopsies, and finally those with polypectomies and biopsies.

Furthermore, examinations were grouped based on gold-standard corrected withdrawal time into three categories; <6 minutes, 6–8 minutes, and >8 minutes, to assess under- and overestimation. The errors between AI and physicians were compared for the cases of faster withdrawal, that is, corrected withdrawal time <6 minutes and under inadequate bowel preparation. In all above cases, measurements were paired, non-normally distributed, and thus compared using the Wilcoxon signed-rank test.

The MAEs for the AI method for examinations in which the beginning of withdrawal was identified via landmarks vs. cases where the beginning of withdrawal was estimated were determined. These unpaired, non-normally distributed values were compared using the Mann–Whitney test. For all measured means, 95%CIs were obtained using bootstrapping as a measure of uncertainty evaluation.

Finally, expert satisfaction with the AI-generated examination report was evaluated. Each participating physician independently reviewed the reports and assessed their quality and clarity using a 4-point Likert-scale questionnaire. To determine consensus and agreement, responses from all evaluators were aggregated for each question. The consistency among the raters was evaluated using interclass correlation coefficients (ICC) 33 .

### Ethics

This study received approval from the medical ethics committee at Julius Maximilian University Würzburg (12/20-am). In alignment with the Helsinki Declaration of 1964 and later versions, signed informed consent was obtained from each patient prior to participation.

## Results

### Recruitment chart and demographics

**Fig. 2:**
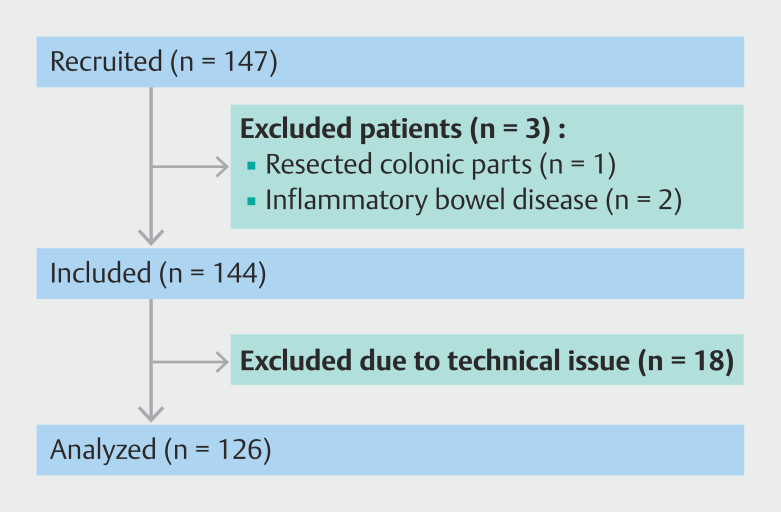
Patient recruitment flow chart for the study.

A total of 147 patients were recruited in a single university hospital in Germany between December 19, 2023 and March 27, 2024. Three patients were excluded due to study exclusion criteria. Eighteen further cases were excluded due a technical issue related to the system on which the AI was running, which failed to store the AI outputs and examination recording (i.e. not due to a technical failure of the AI system). Finally, 126 patients were eligible for analysis. The patient recruitment flow chart for the study is depicted in Fig. 2 . Characteristics of patients and examinations are presented in Table 1 .

**Table:** **Table 1** Characteristics of the examinations included in the study.

Characteristic	N = 126
Sex, n (%)	
Female	64 (50.8)
Male	62 (49.2)
Age, median (Q1–Q3), years	64 (56–71)
Indication, n	
Screening	13
Surveillance	63
Symptomatic	42
Unknown	8
Withdrawal time, median (Q1–Q3), minutes	11.2 (7.1–18)
Corrected withdrawal time, median (Q1–Q3), minutes	10.4 (6.9–14.4)
BBPS, median (Q1–Q3)	9 (7–9)
At least one segment with BBPS <2, n (%)	5 (4.0)
Cecal intubation, n (%)	126 (100)
Corrected withdrawal time, n (%)	
<6 minutes	25 (19.8)
6–8 minutes	19 (15.1)
>8 minutes	82 (65.1)
Procedures undertaken during the exam, n (%)	
No procedures	42 (33.3)
Only biopsies	24 (19.1)
Only resections	34 (27.0)
Resections and biopsies	26 (20.6)
Number of endoscopists	10
BBPS, Boston Bowel Preparation Scale; Q1–Q3, quartile 1–quartile 3.	

### MAE of withdrawal time estimations

**Fig. 3:**
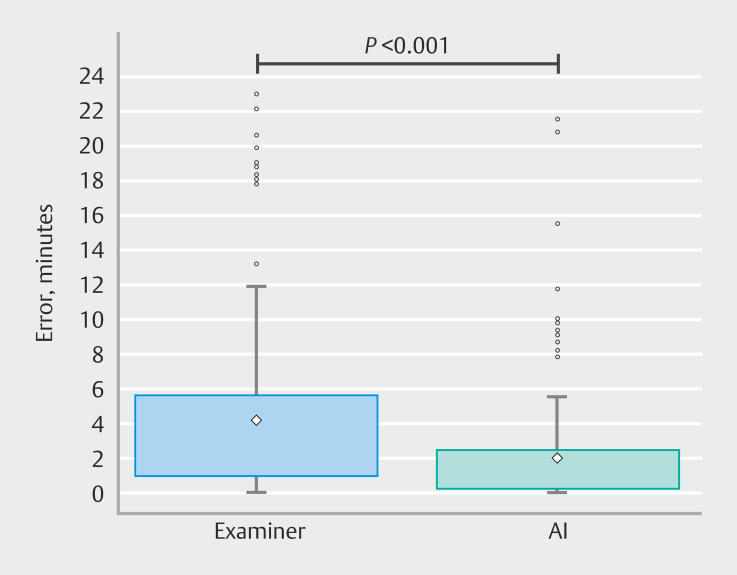
The distribution of absolute errors in minutes for withdrawal time estimation. Diamonds indicate the mean value, box limits correspond to the interquartile range (IQR), whiskers extend to 1.5-times the IQR, and dots indicate points outside the 1.5 times IQR range. AI, artificial intelligence.

**Fig. 4:**
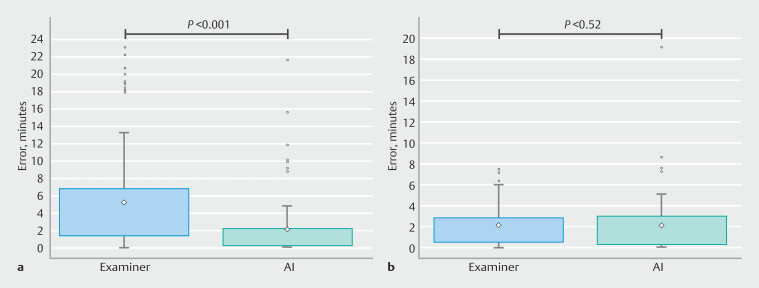
The distribution of absolute errors for physicians (blue) and artificial intelligence (turquoise). **a** Examinations with at least one polyp resection. **b** Examinations with no polyp resections. Diamonds indicate the mean value, box limits correspond to the interquartile range (IQR), whiskers extend to 1.5-times the IQR, and dots indicate points outside the 1.5 times IQR range. AI, artificial intelligence.

The AI system had an MAE of 2.2 minutes (95%CI 1.6–2.8) for withdrawal time estimation, which was significantly lower than the physician MAE of 4.2 minutes (95%CI 3.4–5.2; *P* < 0.001) ( Fig. 3 ). The MAEs of participating physicians are presented in **Table 1s** . The MAE of AI for estimating the beginning of withdrawal was 2.2 minutes (95%CI 1.6–2.9), whereas the MAE when estimating the duration of interventions was 0.9 minutes (95%CI 0.7–1.1). Of the 126 examinations analyzed, 84 (66.6%) included at least one intervention. In these cases, the AI MAE was 2.1 minutes (95%CI 1.4–2.9), which was significantly less than the physician MAE of 5.2 minutes (95%CI 4.0–6.5; *P* < 0.001). In the remaining 42 examinations (33.3%), during which no polypectomy was performed, the AI MAE was 2.3 minutes (95%CI 1.3–3.6), which was comparable to the 2.3 minutes (95%CI 1.6–3.0) MAE for physicians ( *P* = 0.52). The distributions of absolute errors for the two subgroups of examinations are displayed in Fig. 4 .

More explicitly, for examinations where only biopsies were taken, the physician MAE of 4.1 minutes (95%CI 2.1–6.5) was larger but comparable to the 2.9 minutes (95%CI 1.3–5.1) of the AI system ( *P* = 0.13). In examinations that only contained polypectomies, the physician MAE was 4.8 minutes (95%CI 3.1–6.7), which was significantly higher than the AI MAE of 1.6 minutes (95%CI 0.7–2.8; *P* < 0.001). Finally, when both resections and biopsies were undertaken, physicians had a significantly higher error of 6.8 minutes (95%CI 4.5–9.3) compared with 2.0 minutes (95%CI 1.0–3.2) achieved by AI ( *P* < 0.001). The distributions of absolute errors for all categories are presented in **Fig.1s** .

Physicians overestimated the duration in 88% of cases where the corrected withdrawal time was <6 minutes, compared with 36% of cases estimated by AI. When corrected withdrawal time was between 6 and 8 minutes, physicians overestimated 63% and AI overestimated 53%, while underestimation was low for both (16% and 11%). Finally, for examinations with duration >8 minutes, physicians and AI both underestimated 7% of the cases. The over- and underestimations for physicians and AI are presented in **Fig. 2s** . Focusing on shorter examinations, where gold standard corrected withdrawal time was <6 minutes, AI had an MAE of 1.5 minutes (95%CI 0.8–2.4) vs. 2.5 minutes (95%CI 1.7–3.5) for physicians.

The proposed AI method estimated the withdrawal time in all 126 analyzed examinations. However, in 68 of them, the beginning of withdrawal was extrapolated, as the deepest point reached during the examination was not explicitly identified. In these examinations, AI had an MAE of 2.5 minutes (95%CI 1.6–3.6), which was comparable to the MAE of 1.8 minutes (95%CI 1.1–2.7) when the beginning of withdrawal was explicitly identified ( *P* = 0.85) ( **Fig. 3s** ). Furthermore, AI was significantly more accurate than physicians in both cases. When the landmark was identified, the AI MAE of 1.8 minutes was significantly lower than the physician MAE of 4.9 minutes (95%CI 3.6–6.5; *P* < 0.001). In cases where the beginning of withdrawal was estimated by AI, the AI MAE of 2.5 minutes was still significantly higher than the physician MAE of 3.6 minutes (95%CI 2.6–4.6; *P =* 0.01) ( **Fig. 4s** ).

Among the cases for which the physician included a Boston Bowel Preparation Scale score in the report, five had scores lower than 2 for at least one examination segment. For these cases, AI had an MAE of 5.7 minutes (95%CI 1.1–13.4) and physicians had a comparable MAE of 3.4 (95%CI 2.7–4.1; *P* = 0.63).

**Fig. 5:**
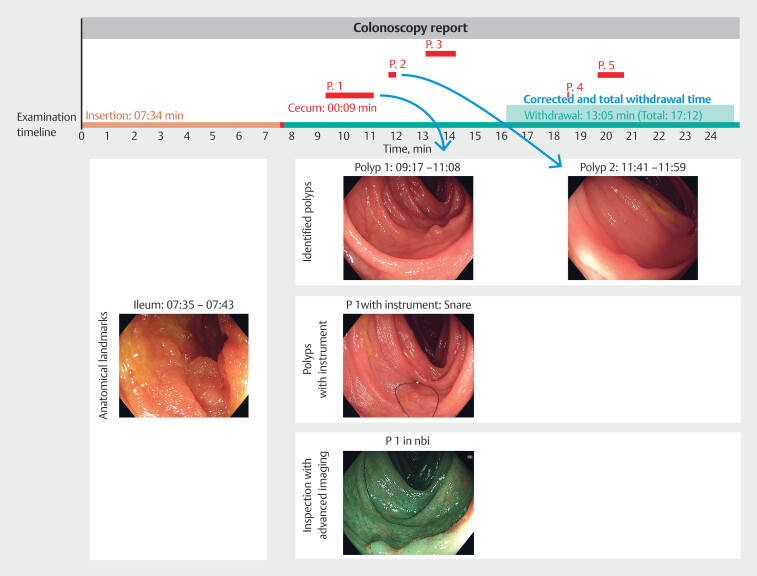
The first page of an image report generated automatically using the artificial intelligence (AI) system. The upper part of the image is the timeline of the examination where the insertion, inspection of the cecum, and withdrawal phases of the colonoscopy and their duration are depicted. Furthermore, each identified polyp is marked above the timeline. On the lower part, images of identified anatomical landmarks are presented in the first column. The second and third columns contain images from polyps identified during the examination, with instruments and advanced imaging when available. Post hoc, blue rectangles and text are added to indicate the locations of the different report elements.

A committee of four endoscopists, each having over 10 years of experience, independently assessed 58 generated image reports that contained images of landmarks identified as the deepest point reached during the examination. The first page of an AI-generated report for the examination is presented in Fig. 5 , along with overlayed annotations for the different report elements. This example page contains images of anatomical landmarks and the first two polyp identifications in the examination. Images of the remaining three polyps identified were contained on the second page of the report, and are not included here for the sake of readability.

The endoscopists appreciated the visualization of examination timelines and case data, with 97% of assessments recorded as satisfactory. The timeline failed to clearly indicate when a polyp was identified in only 5% of cases. Additionally, the corrected withdrawal times were reported to be easily visualized in 98% of assessments. The image quality in the report met satisfactory standards in 79% of instances, and the images detailing the deepest point reached were found to be informative in 76% of cases. For assessing treatment, reviewers felt that 88% of the AI-selected images alone were sufficient for confidently characterizing a polyp using the Paris classification. Moreover, endoscopic instruments used during treatment were not recognizable in only 4% of cases. Overall, for 81% of assessments, reviewers expressed satisfaction with the automatically generated report. The inter-rater consensus coefficient (ICC) was 0.92 (95%CI 0.89–0.95), reflecting excellent consistency among raters. The total expert assessments for the qualitative evaluation are depicted in **Fig. 5s** .

## Discussion

In this prospective superiority trial, we compared an AI system for calculating withdrawal time during screening colonoscopy with physicians’ estimations when performing examinations. The results showed that AI had an MAE of 2.2 minutes, which was significantly lower than the 4.2 minutes of physicians ( *P* < 0.001). This difference can be attributed to examinations where at least one endoscopic intervention was undertaken, where AI MAE was 2.1 minutes, compared with 5.2 minutes for physicians ( *P* < 0.001). Physicians and AI had comparable errors in examinations without intervention. The observed improvement in AI accuracy is not surprising, as precisely estimating the time spent on interventions is the most challenging part of calculating withdrawal time. Analyses revealed that determining the beginning of withdrawal was the biggest challenge for the AI system.

We investigated the impact of interventions on withdrawal time assessment. Physicians struggled more when polypectomies were involved compared with when only biopsies were undertaken (MAE: 4.8 vs. 4.1 minutes). As expected, for examinations that contained both, physicians tended to have higher errors (MAE 6.8 minutes). Conversely, AI tended to be more accurate when resections were performed compared with biopsies (MAE: 1.6 vs. 2.9 minutes), with an MAE of 2.0 minutes when both resections and biopsies were performed. This could be attributed to resections being easier to identify compared with biopsies, due to their faster execution.

Our results also verified the expected result that physicians tend to overestimate withdrawal time. This becomes evident by the overwhelming 88% of examinations with corrected withdrawal time of <6 minutes that were overestimated by physicians. For the same category, AI overestimated 36% of cases, with only 8% of them being identified as >8 minutes. For examinations with withdrawal time <6 minutes, AI demonstrated an MAE of 1.5 minutes, which was lower than the overall error. This could be explained by faster endoscope movements hindering image quality. Even in this case, the AI had a lower error compared with the 2.5 minutes of physicians.

The AI system also automatically generated an image report for the examination, which included a color-coded timeline of the examination phases and indicative images of landmarks or polypectomies undertaken during the examination. The expert evaluation of the 58 reports showed that the reports were intuitive and thorough, with high-quality images. The timeline representation was intuitive and clear in 97% of the evaluations. Furthermore, 79% of the evaluations indicated satisfaction with the quality of the images included. Overall, experts were satisfied with 81% of the reports generated.

In this study, AI withdrawal time estimation was compared only against the manually annotated gold standard. These results were not shown to physicians participating in the study to avoid introducing bias. Furthermore, automatically generated reports were evaluated by physicians in terms of content clarity and completeness. Considering the solid performance of AI, the next step will be to present the results to physicians after the examination to investigate the impact of human–AI interactions.

Withdrawal time has been measured in multiple studies that prospectively evaluated AI use in colonoscopy 34 35 . Yet, to our knowledge, no other prospective studies have evaluated the efficacy and generalizability of AI-based withdrawal time calculation under real-world conditions as the primary outcome. Comparing our results with existing retrospective studies reveals similar trends, with AI demonstrating better accuracy in withdrawal time estimation compared with physician estimations. However, the observed differences in error rates between this study and other works, reporting a median error of 24 28 or 41 seconds 27 , may be attributed to the prospective nature of our trial and the use of out-of-distribution data. Furthermore, a recent study investigated an AI-based report-generation system for colonoscopy, focusing on the time saved by endoscopists when using their proposed method 29 . These findings emphasize the importance of considering the specific context and limitations of each study when interpreting results.

This work is subject to limitations. First, the study was conducted at a single center, which might limit the variability of patients and devices. While we believe that the AI system would generalize well to other settings, further studies are needed to validate this. Second, withdrawal time was estimated according to physicians’ discretion, resulting in non-standardized assessments, which reflects real clinical practice. Furthermore, there were instances where AI failed to accurately identify the deepest point reached during the examination. However, we demonstrated that the error in cases where the deepest point was explicitly identified and those where it was estimated were comparable. This can be further improved by increasing the amount of training data for the AI model and implementing additional methods to improve detection of the deepest examination point. Additionally, most patients analyzed had adequate bowel preparation. As a result, the method was not evaluated in cases of inadequate preparation, which can affect the performance of AI, as shown by the AI having a higher but comparable MAE to that of physicians. However, most patients, especially in the context of screening colonoscopy, typically have adequate bowel preparation. Finally, the AI-generated report only contained the examination timeline and images selected, but not any text. However, the structured format of the AI output can be combined with other technologies, such as large language models, to generate a human-readable report that adheres to formatting limitations of existing clinical data management systems.

In conclusion, our study underscores the significant advantages of AI in clinical workflows, especially for tasks demanding memorization, such as withdrawal time estimation. Our AI system effectively generated image-based reports for colonoscopies, including a detailed timeline and selection of indicative images. Owing to the prospective nature of the study, we are confident that the proposed method could be used in clinical practice without significant alterations. By embedding these capabilities within current reporting systems, there is potential to alleviate the bureaucratic burden on physicians and reduce post-examination tasks. This integration could enhance efficiency and accuracy in clinical settings, ultimately contributing to improved patient care outcomes.

## Supplementary Material

Supplementary Material Zusatzmaterial
