# Supplementary material for: A prospective study evaluating an artificial intelligence-based system for withdrawal time measurement
Source: Endoscopy. 2025 Nov 20;58(4):367–75. doi: 10.1055/a-2721-6798 (PMC13063404; doi:10.1055/a-2721-6798)

Supplementary Material

A prospective study evaluating an artificial intelligence-based system for withdrawal time measurement

Ioannis Kafetzis, Philipp Sodmann, Bianca-Elena Herghelegiu, Michela Pauletti, Markus Brand, Katrin Schöttker, Wolfram G. Zoller, Jörg Albert, Alexander Meining, Alexander Hann

Supplementary methods

Description of the AI-based method for withdrawal time estimation.

The proposed method leverages an AI model trained to identify specific labels within endoscopic images, including pathological findings (such as polyps), endoscopic instruments (e.g. snares, biopsy forceps), and anatomical landmarks (like the ileum, ileocecal valve, and appendiceal orifice). The AI also evaluates image quality and distinguishes between frames captured inside and outside the body. Frames identified as "outside" are automatically excluded from further analysis.

The core of the AI model is based on a ConvNext-nano architecture [1], which extracts image features to feed into a classification head introduced in [2]. For each image, the output is a tensor with 16 elements, representing unique labels and indicating the AI-model's confidence level (ranging from 0 to 1) of label presence in the image.

After obtaining predictions for all frames in an endoscopic video, the post-processing phase initiates. The primary objective here is to detect continuous frame intervals where specific labels are consistently identified; these intervals are termed "segments." To mitigate misclassifications arising from poor-quality images, AI predictions undergo calibration based on AI image quality assessment. Subsequently, a one-dimensional convolution smooths these predictions by integrating data from adjacent frames, enhancing robustness. The smoothed label predictions are then compared against predefined thresholds, which were determined through prior study and preliminary evaluations, to further filter noise. Additional refinement is achieved using binary morphological operations of one-dimensional opening and closing, which eliminate short-lived prediction fluctuations.

Finally, segments are identified by grouping consecutive frames consistently showing a specific label. To pinpoint the start of withdrawal in endoscopy, the algorithm first locates the most recent segment featuring key anatomical landmarks like the ileum, ileocecal valve, or cecum. If standard detection methods fall short, an alternative smoothing-based approach is employed. This involves averaging predictions for these landmarks and applying an exponential moving average to smooth the resulting time series. The highest point in this smoothed series helps identify surrounding inflection points, with the most recent one marking the withdrawal's onset. This refined method ensures a systematic, robust analysis of endoscopic recordings, enhancing diagnostic accuracy through AI-driven insights.

The end of withdrawal is defined as the final frame in which the AI does not predict it to be "outside" the patient's body. Polyp and procedure segments are identified by detecting segments where labels such as polyp, biopsy forceps, snare, and clip are consistently present. These segments help delineate specific procedural events or findings within the endoscopic examination, facilitating a comprehensive understanding of the recorded data. This structured approach not only enhances diagnostic precision but also streamlines the generation of detailed reports from endoscopic recordings through AI-driven analysis.

References

[1] Liu, Z., Mao, H., Wu, C. Y., Feichtenhofer, C., Darrell, T., & Xie, S. (2022). A convnet for the 2020s. In Proceedings of the IEEE/CVF conference on computer vision and pattern recognition (pp. 11976-11986).

[2] Kafetzis I, Fuchs K-H, Sodmann P, et al. Efficient Artificial Intelligence-based Assessment of the Gastroesophageal Valve with Hill Classification through Active Learning. Sci Rep 2024; doi:10.1038/s41598-024-68866-x

Table 1s Analysis of the absolute errors in corrected withdrawal time estimation per participating physician.

	Examinations	Examiner MAE, Minutes (95% CI)	AI MAE, Minutes (95% CI)	p-value
**Examiner 1**	25	5.6 (3.51-7.95)	3.5 (1.65-5.78)	0.087
**Examiner 2**	24	2.9 (1.59-4.71)	1.9 (0.72-3.25)	0.013
**Examiner 3**	23	4.5 (2.47-7.24)	2.5 (0.98-4.69)	0.023
**Examiner 4**	14	6.5 (2.85-10.76)	0.8 (0.32-1.43)	0.002
**Examiner 5**	11	3.0 (1.53-4.78)	2.6 (1.11 4.31)	0.844
**Examiner 6**	9	2.4 (0.85 4.53)	2.1 (0.62 4.31)	0.536
**Examiner 7**	7	4.8 (2.37 7.67)	1.5 (0.45 2.79)	0.128
**Examiner 8**	7	3.1 (2.24 4.16)	1.1 (0.56 1.68)	0.002
**Examiner 9**	5	4.0 (1.77 6.11)	1.0 (0.16 2.29)	0.055
**Examiner 10**	1	0.8	1.1	-

Endoscopy | DOI 10.1055/a-2721-6798 | © The Author(s).

Fig. 1s Comparison of absolute errors in corrected withdrawal time calculation for physicians and AI based on the type of interventions undertaken during the examination.

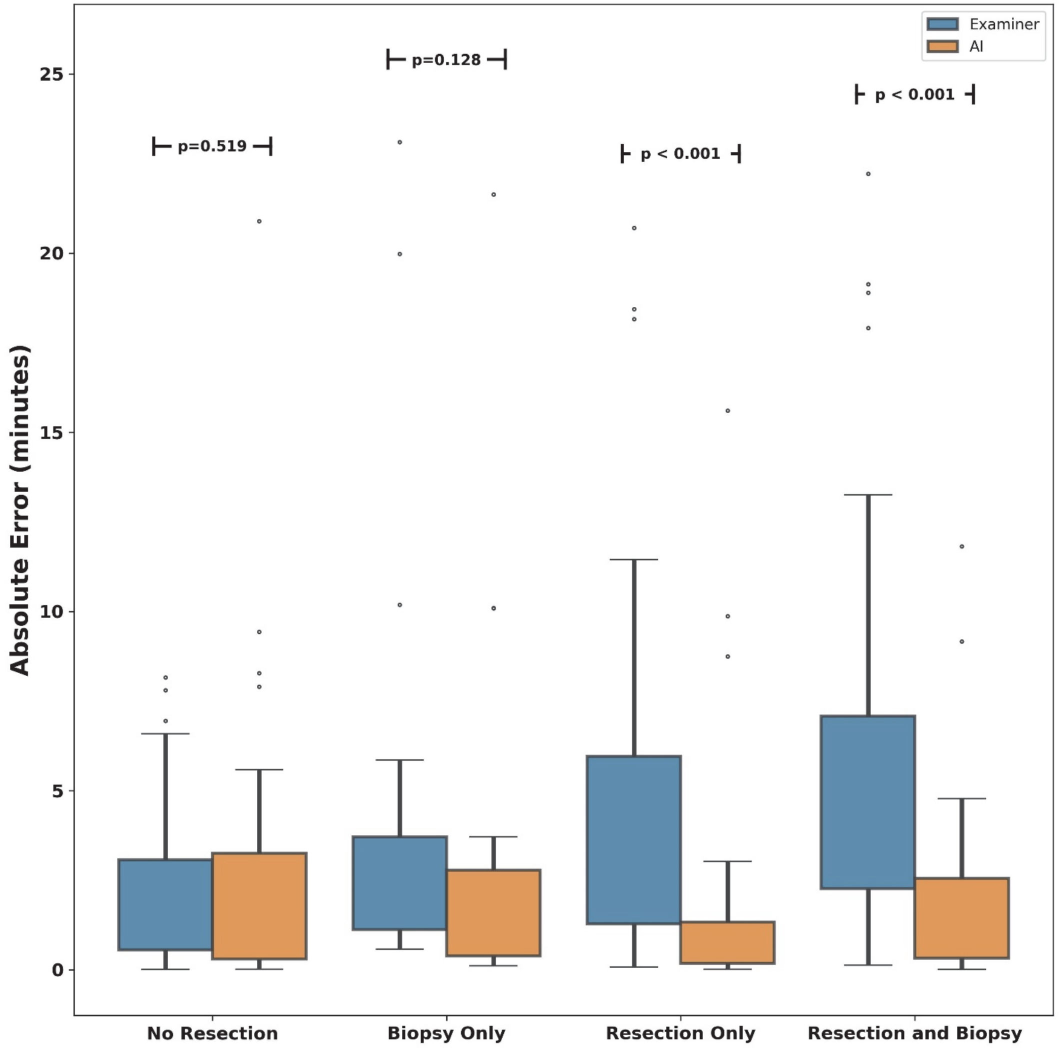

Endoscopy | DOI 10.1055/a-2721-6798 | © The Author(s).

Fig. 2s Over- and underestimations of the corrected withdrawal time from physicians and AI.

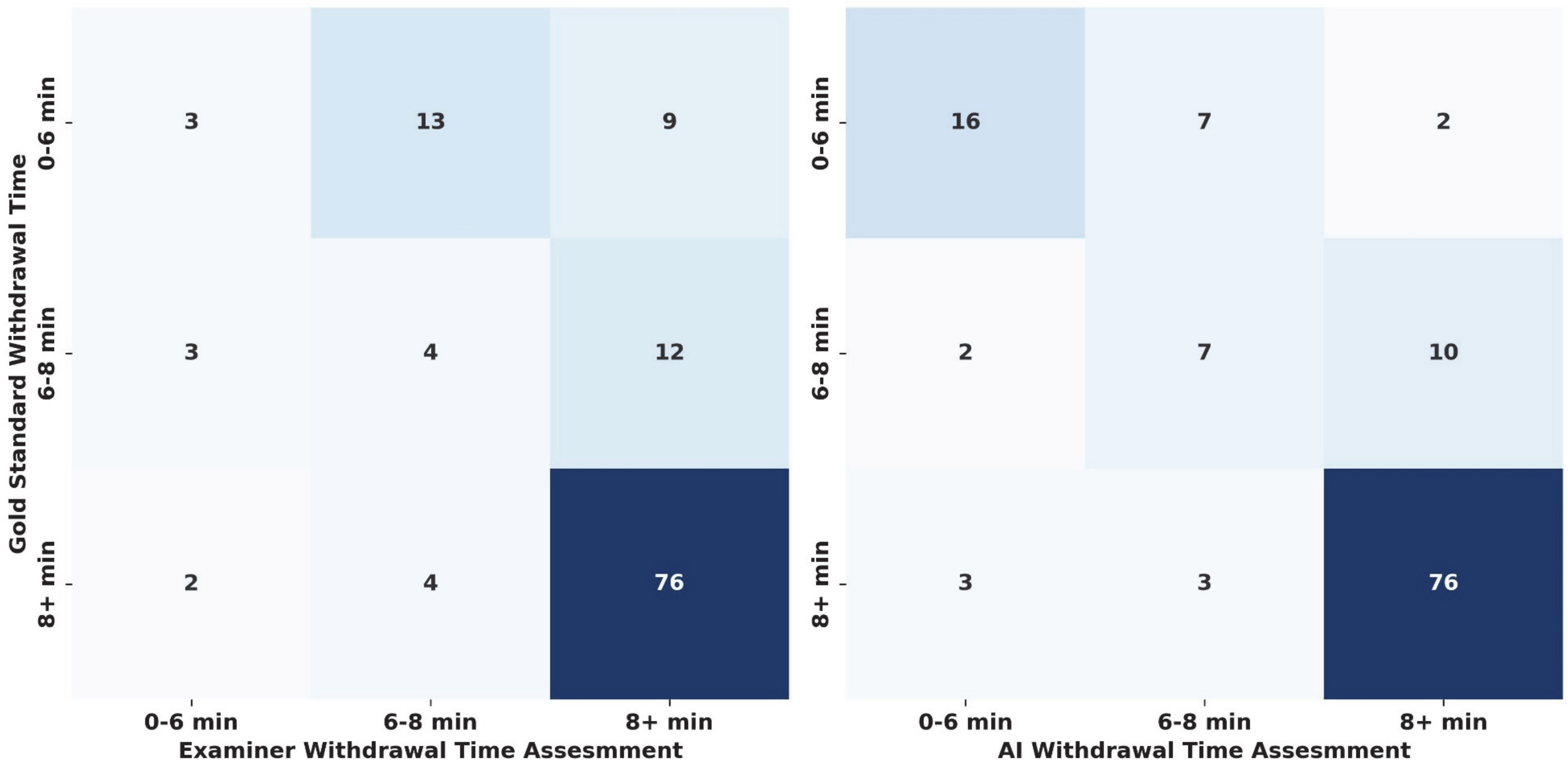

Fig. 3s Distribution of absolute errors of withdrawal time for the AI method when the beginning of withdrawal was explicitly identified (blue) versus when the beginning of withdrawal was estimated.

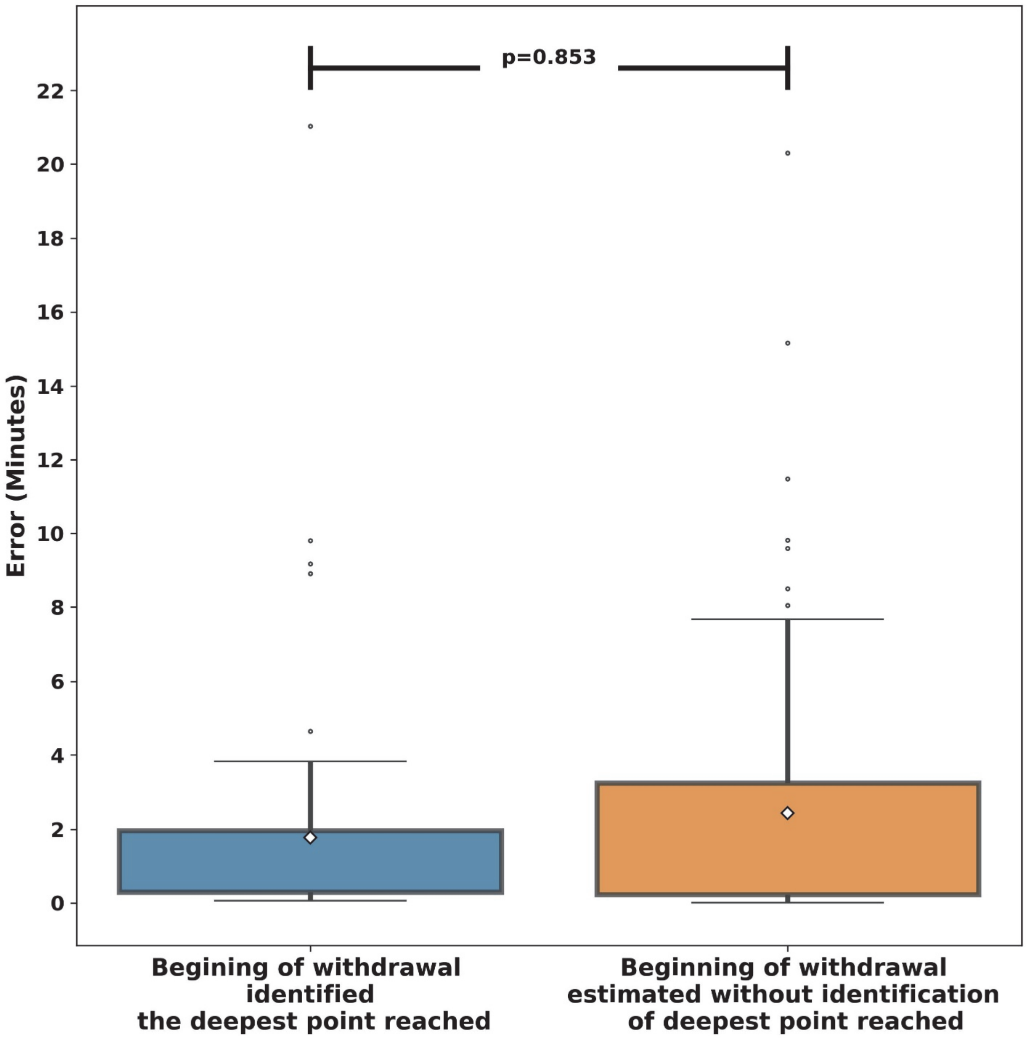

Fig. 4s Comparison between the examiner and AI absolute error for examinations where the beginning of withdrawal time was determined based on identification of an anatomical landmark (left) and the cases where the AI estimated the beginning of withdrawal (right).

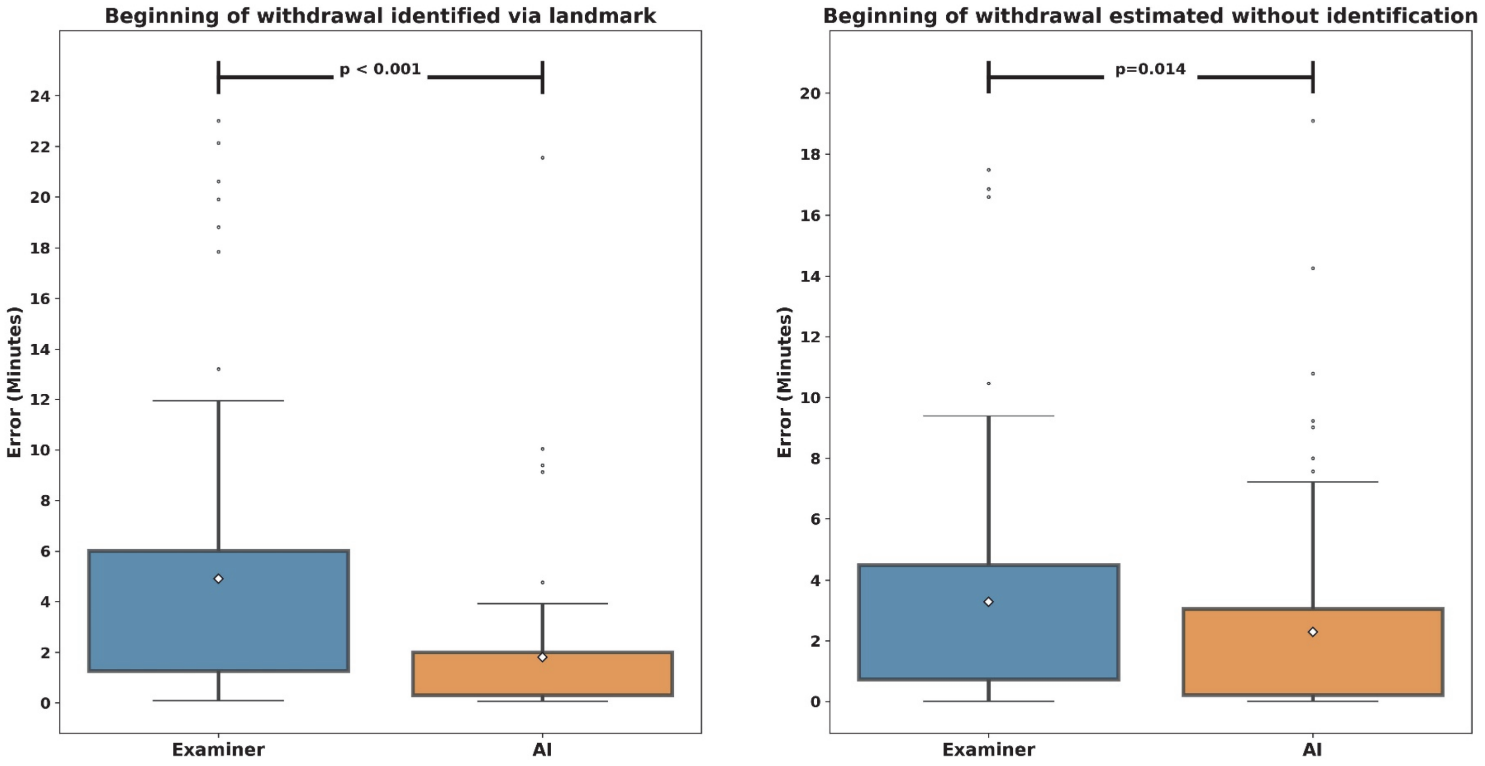

Fig. 5s Results of expert assessment of clarity and quality of the automatically generated image reports. Presented results are obtained from the cases where the questions were applicable.

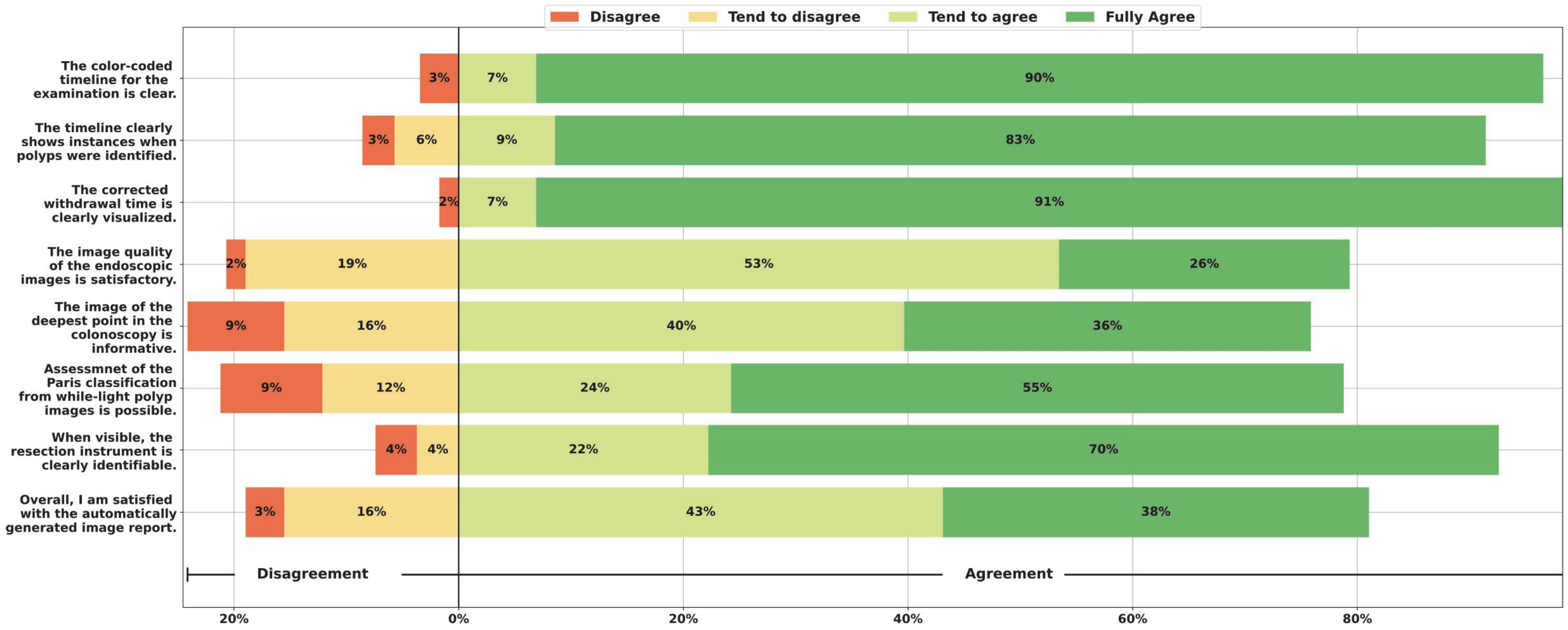

Supplement: Supplementary file 1 — Supplementary Material [file 10-1055-a-2721-6798_27369106.pdf]
